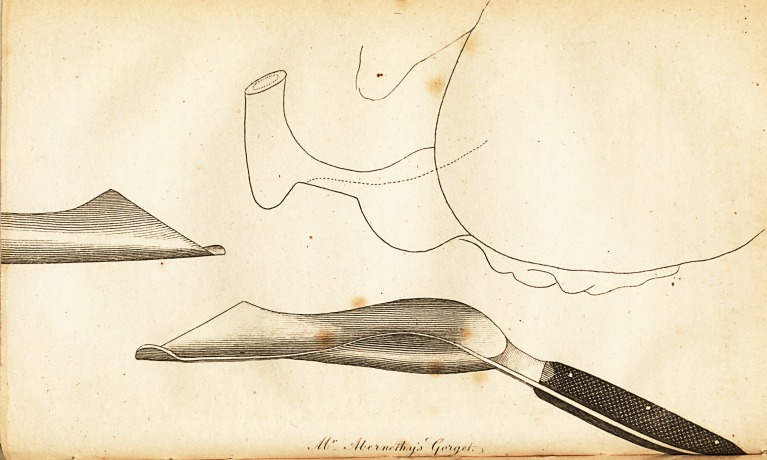# Mr. Abernethy, on the Gorget

**Published:** 1803-05-01

**Authors:** J. Abernethy


*Mr. Abernethy's Gorget.*

THE

### Medical and Phyfical Journal.

VOL. IX.] MAY 1, 1803. [NO. LI.

*Printed for R. PHILLIPS, by W. Thorne, Red Lion Court, Fleet Street, London.*

### To Dr. BATTY.

SIR,

I HAVE felt a little vexation of late from hearing that Mr. Abernethy's gorget for Lithotomy was inquired for in the instrument shops; and still more from finding that an ill contrived instrument, under that appellation, was propofed to be sent into the country.

I wish to disclaim all merit as the inventor of such an instrument, and to state to Surgeons in general, through the medium of your useful and widely circulated publication, the circumstances which probably gave rise to the inquiry for an instrument of my invention. From the commencement of my Lectures on the Operations of Surgery, I have been accustomed to say to the students, that surgeons seemed to me to be too fond of inventing and using new instruments; that the way to appreciate the perfection of an instrument was, to enquire what was wished to be accomplished by it, and if this was effected, it was absurd to change it for another.

If the urethra, prostate gland, and front of the bladder are designed to be divided on one side only, the best direction of the wound appears to me to be between the horizontal and perpendicular directions, in the manner represented in the plate; for a wound thus situated is more likely to heal speedily, from the urine flowing less frequently through it; the prostate is also less injured, but little of its substance being cut through, and the consequent inflammation being less likely to affect its own excretory ducts, or those of the seminal organs. Hawkins's gorget divides the parts in this direction, but not perfectly; for as the cutting edge is curved, partly extending horizontally, and gradually approaching towards the perpendicular, it must make an haggled wound, as will be more easily conceived than described. To remedy this defect, I

recommended that the cutting edge of the gorget should in every part have the same direction, and be turned up at an angle of 45 degrees, as represented in the plate. The back part of the edge will of course pass through a wound made by the front, and from its obliquity it will cut with great facility. The edge of Hawkins's gorget also makes a wound of too small extent. This, surgeons in general perceived, and in avoiding one error, they, as is common, ran into the opposite extreme, and caused their gorgets to be made with lancet shaped processes, which extended so far from the beak, that it must be difficult to get them beyond the bone into the pelvis, and, under these circumstances, it was almost impossible that the pudendal artery should escape division. Three quarters of an inch is the distance to which I have directed the cutting edge to extend from the beak in a gorget for operating on the adult subject.

This instrument will make such a division of the membranous part of the urethra, prostate and anterior part of the bladder, as is represented in the plate; it will not touch the bone, nor divide the pudendal artery. Should the stone be of extraordinary magnitude, I would recommend a surgeon to introduce his finger into the bladder, and divide the front of it with a probe-pointed knife, a little further than the gorget has done, and in the same direction, and, I think, the wound will then easily stretch to an extent equal to the transverse space existing between the rami of the iscium. Indeed, stones sometimes occur of such magnitude that it appears wrong to attempt their extraction entire; as it cannot be done without much injury not only to the bladder, but also to the contiguous parts.

In Hawkins's gorget, which was designed as an improvement upon the blunt one, the form of the latter is still preserved; the sides of the instrument extending in both directions to equal distances from the beak. The blunt side, unless the instrument be much turned awry, must consequently injure the undivided part of the urethra. I therefore recommended that it should be filed away, as is represented in the plate. Again, as it appears of consequence that the cutting edge of the gorget should come in exact contact with the groove of the staff, in whatever direction the one instrument be held with respect to the other, I have always desired that the beak should be ground into an exact segment of a circle, the radius of which should be precisely the same with the depth of the groove of of the staff. This circumstance is also sketched out on the plate.

Such, Sir, are the alterations, which I recommended to be made in Hawkins's gorget, and such is the instrument which, perhaps from an improper dislike to innovations, I shall continue myself to use till I find some fault in it. I have extracted as large stones through the wound made by it, as I should conceive myself warranted in extracting entire, and I have never divided with it the pudendal artery.* As this instrument exactly accomplishes what it is designed to execute, I consider it as perfect of its kind; but it is not my intention to recommend it in preference to other gorgets.

A gentleman,† whose professional knowledge and opportunities qualify him to decide on such subjects, has of late recommended that both sides of the urethra and prostate should be divided, in order to facilitate the extraction of the stone.

I have sent you these remarks merely because I thought they might be of some utility, by inducing surgeons, who have not those extensive opportunities of surgical and anatomical observations, which occur in a metropolis, to reflect on the objects designed to be effected by gorgets for lithotomy in general, and the principles on which they should be constructed; and by these means to guard against the ill consequences which might ensue from the use of improper gorgets obtruded on them by ignorant makers of such instruments.

I am, &c. &c.

*April* 16, 1803. J. ABERNETHY.

---

* It may perhaps be useful to mention, that when I once saw the pudendal artery divided in the adult, the hæmorrhage was so profuse as to call for some sudden mode for suppressing it; I therefore, with my finger, compressed the trunk of that vessel, as it passes along the inner surface of the tuberosity and ramus of the iscium. This pressure served, like the tourniquet, to suppress the bleeding; but on the least remission, the blood gushed from beneath the os pubis so vehemently, and from such a depth, as to make it unlikely that the divided artery could be secured in that situation; I therefore tied the trunk of the vessel where I had compressed, and where it was easily accomplished, and thus effectually prevented any further loss of blood.

† Mr. Astley Cooper.

## Figures and Tables

**Figure f1:**